# Long-term efficacy and safety of tenofovir disoproxil fumarate in Chinese patients with chronic hepatitis B: 5-year results

**DOI:** 10.1007/s12072-019-09943-6

**Published:** 2019-04-11

**Authors:** Xieer Liang, Zhiliang Gao, Qing Xie, Jiming Zhang, Jifang Sheng, Jun Cheng, Chengwei Chen, Qing Mao, Wei Zhao, Hong Ren, Deming Tan, Junqi Niu, Shijun Chen, Chen Pan, Hong Tang, Hao Wang, Yimin Mao, Jidong Jia, Qin Ning, Min Xu, Shanming Wu, Jun Li, Xinxin Zhang, Wenyan Zhang, Cui Xiong, Jinlin Hou

**Affiliations:** 1 State Key Laboratory of Organ Failure Research, Guangdong Provincial Key Laboratory of Viral Hepatitis Research, Department of Hepatology Unit and Infectious Diseases, Nanfang Hospital, Southern Medical University, Guangzhou, 510515 China; 2 3rd Affiliated Hospital of Sun Yat-Sen University, Guangzhou, China; 3 Ruijin Hospital Affiliated to Jiaotong University, School of Medicine, Shanghai, China; 4 Huashan Hospital Affiliated to Fudan University, Shanghai, China; 5 1st Affiliated Hospital of ZheJiang University, Hangzhou, China; 6 Beijing Ditan Hospital, Capital Medical University, Beijing, China; 7 Shanghai the 85th Hospital Affiliated to Nanjing Military, Shanghai, China; 8 Southwest Hospital, Army Medical University, Chongqing, China; 9 2nd Hospital of Nanjing, Nanjing, China; 10 2nd Affiliated Hospital Chongqing Medical University, Chongqing, China; 11 Xiangya Hospital Central-South University, Changsha, China; 12 1st Affiliated Hospital of Jilin University, Changchun, China; 13 Jinan Hospital for Infectious Disease, Jinan, China; 14 Mengchao Hepatobiliary Hospital of Fujian Medical University, Fujian Sheng, China; 15 West China Hospital, Sichuan University, Chengdu, China; 16 Peking University People’s Hospital, Beijing, China; 17 RenJi Hospital, Shanghai Jiaotong University School of Medicine, Shanghai, China; 18 Beijing Friendship Hospital, Capital University, Beijing, China; 19 Tongji Hospital, Tongji Medical College, Huazhong University of Science and Technology, Wuhan, China; 20 Guangzhou Eighth Municipal People’s Hospital, Guangzhou, China; 21 Shanghai Public Health Clinical Center, Shanghai, China; 22 The First Affiliated Hospital with Nanjing Medical University, Nanjing, China; 23 GlaxoSmithKline R&D Company Limited, Shanghai, China

**Keywords:** Antiviral therapy, Chronic hepatitis B, Long-term tenofovir disoproxil fumarate, Virological suppression

## Abstract

**Background and aim:**

Long-term treatment with tenofovir disoproxil fumarate (TDF) has demonstrated suppression of viral replication outside of China. This study aims to assess efficacy, resistance and safety of TDF for up to 240 weeks in Chinese patients with chronic hepatitis B virus (HBV) infection.

**Methods:**

Patients (HBeAg-positive or HBeAg-negative) who were randomised to receive TDF 300 mg or adefovir dipivoxil (ADV) 10 mg once daily in the 48-week double-blind phase (*N* = 498) were eligible to enter the open-label TDF phase (TDF–TDF and ADV–TDF groups) for additional 192 weeks.

**Results:**

Overall, 457/512 (89.3%) randomised patients completed 240 weeks of treatment. Virological suppression was achieved in 84.5% and 87.9% in HBeAg-positive patients and 89.6% and 89.5% in HBeAg-negative patients in TDF–TDF and ADV–TDF groups, respectively, at week 240. The majority of patients from both groups had normalized alanine transaminase levels. More patients had HBeAg loss (41.7% vs. 36.4%) and HBeAg seroconversion (32.0% vs. 28.3%) in TDF–TDF than in ADV–TDF group, respectively. Only one HBeAg-positive patient in TDF–TDF group had HBsAg loss at week 240. No evidence of resistance to TDF was observed. The incidence of adverse events was similar in both groups (TDF–TDF, 56.4% vs. ADV–TDF, 51.6%). One patient had serum creatinine elevation ≥ 0.5 mg/dL above baseline, and three patients had confirmed grade 3/4 phosphorus abnormalities (< 2 mg/dL).

**Conclusion:**

In Chinese patients with chronic HBV, long-term treatment with TDF showed sustained viral suppression without development of resistance up to 240 weeks. No new safety concerns were found with TDF in this patient population.

*Clinical Trial Registration* ClinicalTrial.gov Identifier NCT01300234; GSK Clinical Study Register 114648.

**Electronic supplementary material:**

The online version of this article (10.1007/s12072-019-09943-6) contains supplementary material, which is available to authorized users.

## Introduction

Infection with hepatitis B virus (HBV) remains a global health problem. In China, 7.1% of the adult population is infected with HBV [1]. Elevated HBV DNA levels are associated with increased risk of cirrhosis and hepatocellular carcinoma (HCC). Therefore, the primary goal of treatment is to attain sustained virological suppression after long-term treatment with antiviral agents, as this has been associated with fibrosis regression and reversal of cirrhosis [1].

Tenofovir disoproxil fumarate (TDF) is a selective inhibitor of HBV DNA polymerase reverse transcriptase currently approved in the USA, Europe, Canada and several Asia Pacific countries, including China, for the treatment of chronic HBV infection. Although long-term clinical data of treatment in patients with HBV are available for global population [2], longer term data in Chinese patients with TDF are not available to the best of our knowledge.

The findings from the 48-week double-blind study demonstrated superiority of TDF over ADV in suppressing HBV DNA levels without development of resistance mutations in Chinese patients with chronic HBV [3]. In this paper, the efficacy and safety results for up to 240 weeks are presented.

## Methods

### Study design and patients

This report pertains to the 192-week open-label phase following the initial 48-week randomised (1:1), double-blind, double-dummy, active-controlled phase of the study comparing TDF 300 mg once daily (o.d.) with ADV 10 mg o.d. conducted in China between March 2011 and December 2016. The detailed study design and results of the 48-week double-blind phase have been reported earlier [3]. Patients who completed the initial 48 weeks of treatment were eligible to receive TDF monotherapy during the open-label phase and were assigned to two groups (TDF–TDF and ADV–TDF) for additional 192 weeks.

The study population has been previously described [3]. Briefly, patients (aged 18 to 69 years) with chronic HBV alanine aminotransferase levels ≥ 2 × upper limit of normal (ULN) in HBeAg-positive patients and > 1× ULN in HBeAg-negative patients, HBV DNA > 10^5^ copies/mL and had not received prior nucleoside or nucleotide treatment (previous lamivudine treatment was allowed in ≤ 10% of the total study population) were included in the study.

Efficacy and safety assessments were performed every 4 weeks for the first 12 weeks and every 12 weeks thereafter, up to 240 weeks. Liver biopsy was performed in a subset of patients at baseline, week 48 and week 240.

The study protocol was approved by an independent ethics committee or institutional review board at each study site. The study was conducted in accordance with the ethical principles laid down in the Declaration of Helsinki. Patients provided written informed consent for the open-label phase at the time of enrollment in the double-blind phase.

### Study assessments

Efficacy endpoints at week 240 included virological response defined as proportion of patients with HBV DNA < 400 copies/mL and log_10_ reduction in HBV DNA, biochemical response defined as proportion of patients with ALT normalization and HBeAg and HBsAg loss and seroconversion. Other assessments were virological breakthrough (defined as an HBV DNA increase of 1 log_10_ copies/mL above the treatment nadir, confirmed on two consecutive visits or last on-treatment measurement), histological improvement (reduction of ≥ 2 points in Knodell necroinflammatory score with no increase in fibrosis) and resistance mutations in HBV pol/RT. Additionally, a subgroup analysis was performed in high viral load (HVL) patients (baseline HBV DNA > 9 log_10_ copies/mL [5.82 copies/mL = 1 IU/mL]) and non-HVL (baseline HBV DNA ≤ 9 log_10_ IU/mL) patients. Treatment compliance was assessed by standard pill counts for patients who returned all unused medications (including empty containers). The returned tablets were counted and compared with the number of tablets expected to be used during the treatment period. Compliance assessment > 80% was considered as compliance to treatment.

Resistance surveillance including genotypic analysis of HBV polymerase gene was performed annually until week 240 when patients had HBV DNA ≥ 400 copies/mL, had confirmed virological breakthrough or remained viraemic due to discontinuation of treatment.

Safety assessments included adverse events (AEs), serious AEs (SAEs), hematology and clinical chemistry parameters. In a subgroup analysis, renal safety was assessed in patients with impaired renal function versus normal renal function, and patients with hypertension (HTN) or diabetes mellitus (DM) at baseline. Predicted HCC incidence was calculated using REACH-B model [4]. Post hoc analyses including REACH-B model, comparison with data from global phase III pivotal studies, Knodell necroinflammatory scores and Knodell fibrosis scores and the demographic characteristics and baseline disease and on-treatment response characteristics associated with reversal or lack of reversal of cirrhosis at year 5 were also performed.

### Statistical analysis

All efficacy assessments including virological, biochemical and serologic endpoints up to 240 weeks were summarized descriptively. A ‘non-completers equal failures’ approach was used for the efficacy analyses in overall population as well as subgroup of HVL and non-HVL patients.

Efficacy analyses were performed using intent-to-treat (ITT) population that included all randomised patients who received ≥ 1 dose of the study medication and per-protocol population in the whole 5 years’ period (PPW) that included patients in the ITT population excluding patients with major protocol deviations in the entire study period of 5 years and early withdrawal from the study.

The safety population included all patients who received ≥ 1 dose of TDF and ADV and had ≥ 1 post-baseline safety assessment. Safety data were summarized descriptively.

## Results

**Fig. 1:**
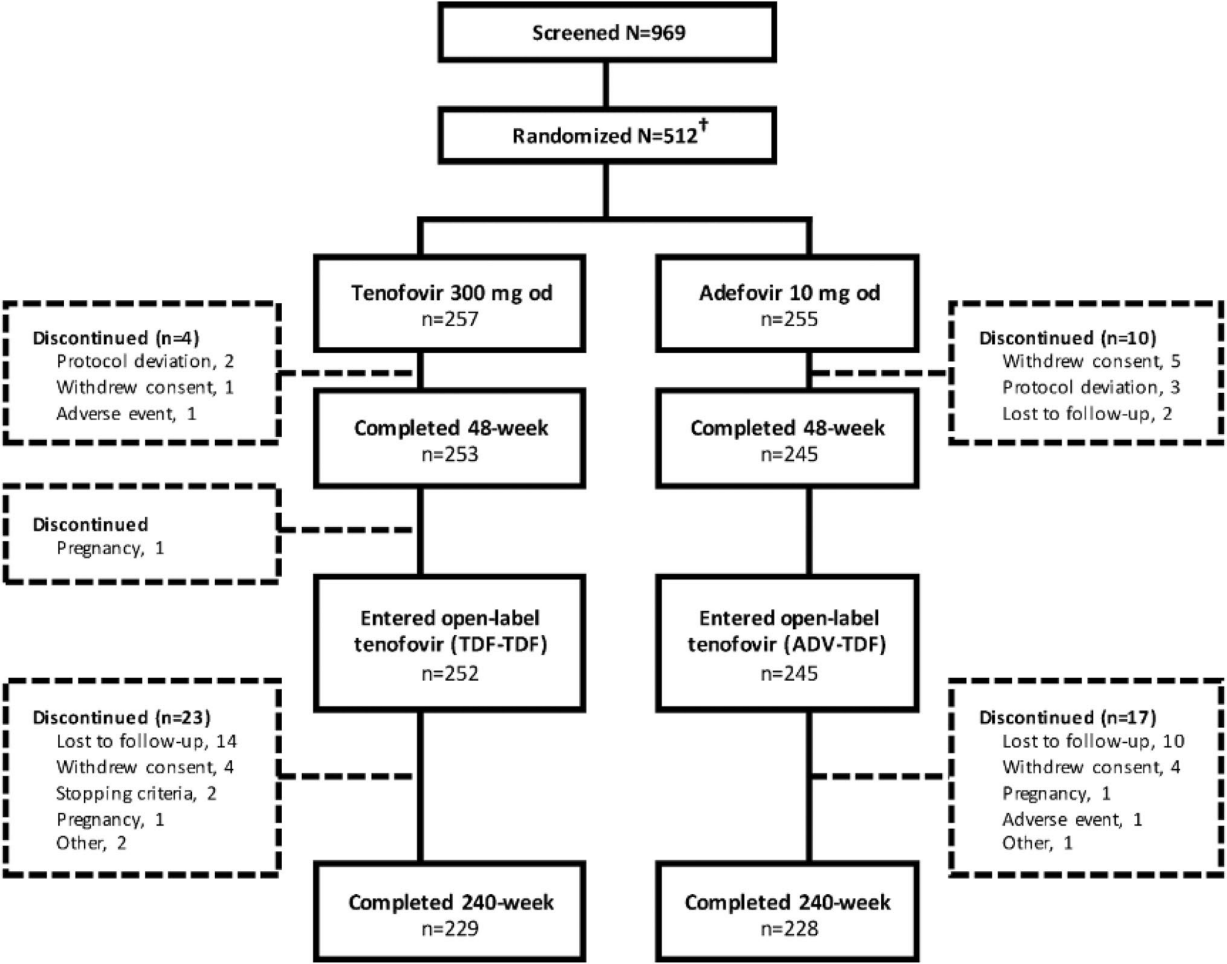
Patient disposition. ADV, adefovir dipivoxil; o.d., once daily; TDF, tenofovir disoproxil fumarate. ^†^Three patients randomised to adefovir 10 mg group did not receive study treatment; hence, total safety and intent-to-treat population was *N* = 509

In total, 512 patients were randomised to TDF 300 mg or ADV 10 mg group, of which three patients randomised to ADV 10 mg group did not receive study treatment. Of the 509 patients, 498 patients completed the 48-week double-blind phase and 497 (99.7%) entered the open-label phase of the study. A total of 457 patients completed the study (Fig. 1). The demographics and baseline characteristics were similar between TDF and ADV groups (Table 1).

**Table 1:** Patient demographics and baseline characteristics (ITT population)

Characteristic	HBeAg-positive		HBeAg-negative	
	TDF (*N* = 103)	ADV (*N* = 99)	TDF (*N* = 154)	ADV (*N* = 153)
Age (years)	30.5 (8.88)	30.3 (7.99)	39.9 (9.76)	40.4 (9.90)
Gender, *n* (%)				
Men	87 (84.5)	81 (81.8)	127 (82.5)	129 (84.3)
Women	16 (15.5)	18 (18.2)	27 (17.5)	24 (15.7)
Ethnicity, *n* (%)				
Asian–East Asian	103 (100.0)	99 (100.0)	154 (100.0)	153 (100.0)
BMI (kg/m^2^)	22.3 (3.22)	22.5 (3.30)	23.3 (2.98)	23.0 (3.03)
HBV DNA (log_10_ copies/mL)	8.7 (0.87)	8.7 (0.79)	6.9 (1.18)	7.0 (1.13)
ALT (U/L)	199.1 (132.81)	189.0 (121.54)	133.4 (120.95)	112.6 (80.35)
PLT (10^9^/L)	169.5 (50.9)	172.4 (47.2)	167.8 (43.8)	161.1 (47.1)
Knodell necroinflammatory score^a^	8.2 (3.48)	8.5 (2.56)	6.9 (2.72)	7.2 (3.08)
Previous treatment with lamivudine, *n* (%)				
Yes	4 (3.9)	4 (4.0)	7 (4.5)	4 (2.6)
No	98 (95.1)	95 (96.0)	146 (94.8)	147 (96.1)
HBV genotype, *n* (%)				
B	49 (47.6)	45 (45.5)	71 (46.1)	74 (48.4)
B/C	3 (2.9)	4 (4.0)	1 (0.6)	0
C	51 (49.5)	50 (50.5)	81 (52.6)	78 (51.0)
Duration of hepatitis B (months), median (range)	97.4 (8–413)	114.0 (7–338)	129.6 (7–429)	139.2 (7–466)

Data are presented as mean (SD) unless otherwise stated. *ADV* adefovir dipivoxil; *ALT* alanine aminotransferase; *BMI* body mass index; *HBV* hepatitis B virus; *ITT* intention-to-treat; *TDF* tenofovir disoproxil fumarate

Baseline HBsAg level, mean (SD): Genotype B: 3.5 log_10_ IU/mL (0.87) Genotype C: 3.4 log_10_ IU/mL (0.59)

^a^For HBeAg-positive patients, *n* = 40 for TDF and *n* = 49 for ADV; for HBeAg-negative patients, *n* = 46 for TDF and *n* = 51 for ADV

### Virological and histological response

**Fig. 2:**
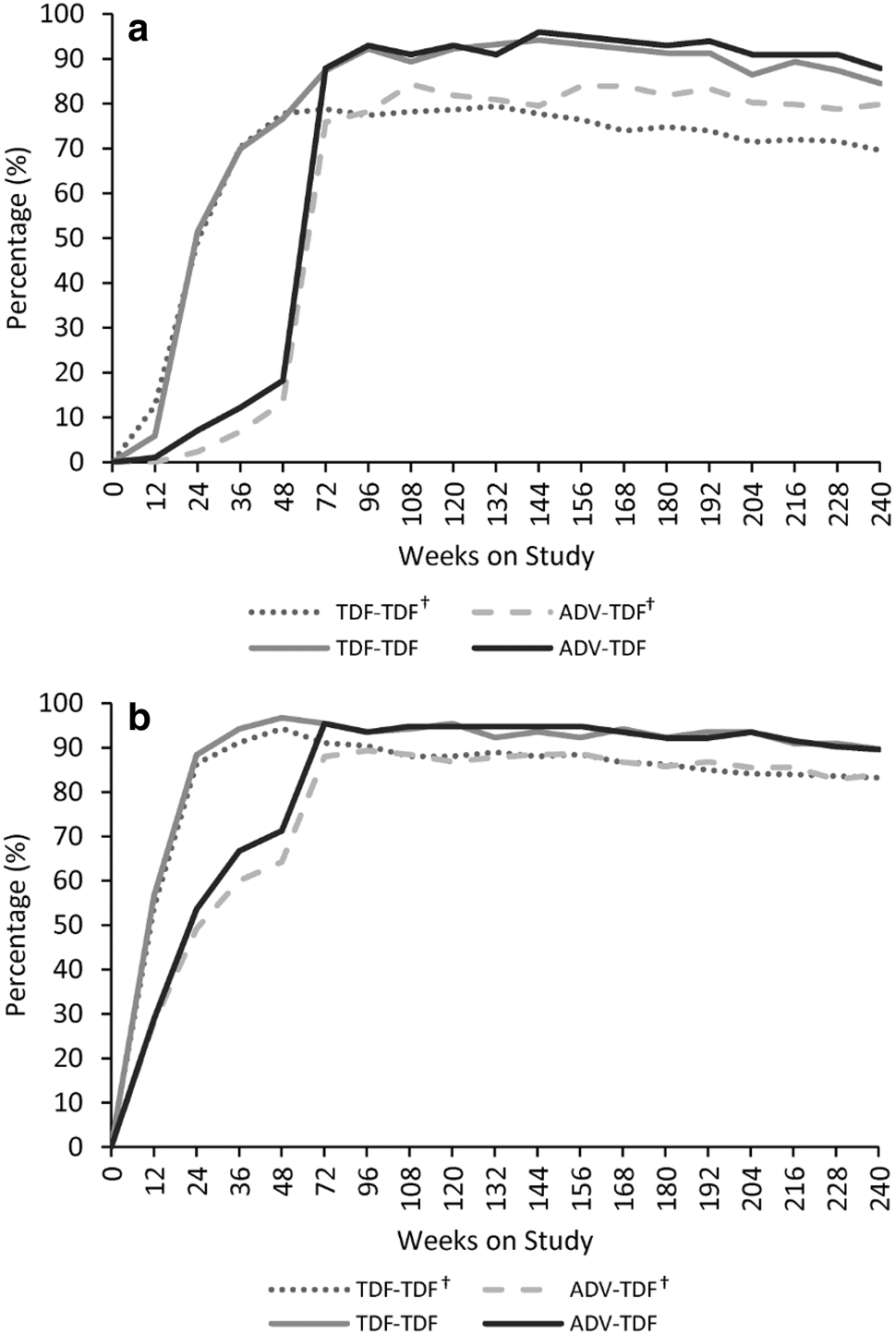
Proportion of patients with HBV DNA < 400 copies/mL over 240 weeks of treatments (ITT). **a** HBeAg-positive, **b** HBeAg-negative. ADV, adefovir dipivoxil; HBV, hepatitis B virus; ITT, intent-to-treat; TDF, tenofovir disoproxil fumarate. ^†^Data on file of global phase III pivotal studies [2, 5]

The proportion of patients achieving HBV DNA levels < 400 copies/ml in the ITT population after 240 weeks was 84.5% and 87.9% in HBeAg-positive patients and 89.6% and 89.5% in HBeAg-negative patients in TDF–TDF and ADV–TDF groups, respectively (Table 2). The overall virological suppression after 240 weeks was slightly higher in the present study than compared with previous pivotal studies (69.6% and 79.8% in HBeAg-positive patients and 83.2% and 83.9% in HBeAg-negative patients in TDF–TDF and ADV–TDF groups, respectively) (Fig. 2) [2, 5].

**Table 2:** Summary of results of efficacy assessments at week 240

	ITT population (*N* = 509)		PPW population (*N* = 444)	
	TDF–TDF	ADV–TDF	TDF–TDF	ADV–TDF
HBeAg-positive, *N*	103	99	89	87
Virological, *n* (%)				
HBV DNA < 69 IU/mL	87 (84.5)	87 (87.9)	85 (95.5)	85 (97.7)
Log_10_ copies/mL reduction in HBV DNA^a^, mean (SD)	− 6.6 (1.01)	− 6.5 (0.79)	− 6.5 (1.01)	− 6.6 (0.77)
Virological breakthrough	4 (3.9)	11 (11.1)	3 (3.4)	7 (8.0)
ALT normalization, *n* (%)	82/102 (80.4)	80/97 (82.5)	81/88 (92.0)	78/85 (91.8)
Serologic, *n* (%)				
HBeAg loss	43 (41.7)	36 (36.4)	41 (46.1)	34 (39.1)
HBsAg loss	1 (1.0)	0	1 (1.1)	0
HBeAg seroconversion^b^	33 (32.0)	28 (28.3)	31 (34.8)	27 (31.0)
HBsAg seroconversion	0	0	0	0
Log_10_ IU/mL reduction in quantitative HBsAg^a^, mean (SD)	− 0.9 (0.97)	− 0.7 (0.91)	− 0.9 (0.98)	− 0.8 (0.92)
HBeAg-negative, *N*	154	153	135	133
Virological, *n* (%)				
HBV DNA < 69 IU/mL	138 (89.6)	137 (89.5)	135 (100.0)	132 (99.2)
Log_10_ copies/mL reduction in HBV DNA^a^, mean (SD)	− 4.9 (1.16)	− 4.9 (1.07)	− 4.9 (1.16)	− 4.9 (1.07)
Virological breakthrough	3 (1.9)	8 (5.2)	1 (0.7)	4 (3.0)
ALT normalization, *n* (%)	119/136 (87.5)	111/132 (84.1)	116/119 (97.5)	107/116 (92.2)
Serologic, *n* (%)				
HBsAg loss	0	0	0	0
HBsAg seroconversion	0	0	0	0
Log_10_ IU/mL reduction in quantitative HBsAg^a^, mean (SD)^c^	− 0.4 (0.62)	− 0.4 (0.51)	− 0.3 (0.62)	− 0.4 (0.51)

Data are presented as *n* (%) unless otherwise stated. *ADV* adefovir dipivoxil; *ALT* alanine aminotransferase; *HBV* hepatitis B virus; *HBsAg* hepatitis B surface antigen; *ITT* intention-to-treat; *PPW* population, per-protocol population in whole 5 years’ period; *SD* standard deviation, *TDF* tenofovir disoproxil fumarate

^a^In the ITT population, HBeAg-positive patients: TDF–TDF (*n* = 91) and ADV–TDF (*n* = 90); HBeAg-negative patients: TDF–TDF (*n* = 138) and ADV–TDF (*n* = 138)

^b^Genotype B patients: 32.98%; Genotype C patients: 28.71%

^c^Mean change (SD) in HBsAg level: Genotype B: − 0.8 log_10_ IU/mL (0.87); Genotype C: − 0.3 log_10_ IU/mL (0.57)

The mean change in HBV DNA levels from baseline in the ITT population was similar in patients receiving TDF–TDF and ADV–TDF at week 240 (− 6.6 log_10_ copies/mL vs. − 6.5 log_10_ copies/mL in HBeAg-positive patients and − 4.9 log_10_ copies/mL in both groups in HBeAg-negative patients). Among patients treated with TDF–TDF in the ITT population, the virological breakthrough rate was numerically lower in both HBeAg-positive and HBeAg-negative patients than ADV–TDF group (HBeAg-positive: 3.9% vs. 11.1%; HBeAg-negative: 1.9% vs. 5.2%). The results for these virological endpoints were similar in the ITT population and PPW population (Table 2). All these patients had > 89% treatment compliance by pill count. Overall, 26 patients experienced virological breakthrough with a median HBV DNA level (range) of 5.54 (3.12–9.04) log_10_ copies/mL. The patients with virological breakthrough continued TDF treatment without adding or switching to other antiviral agents at the discretion of investigator as viral suppression was subsequently observed at the follow-up visits on continuing treatment with TDF.

**Fig. 3:**
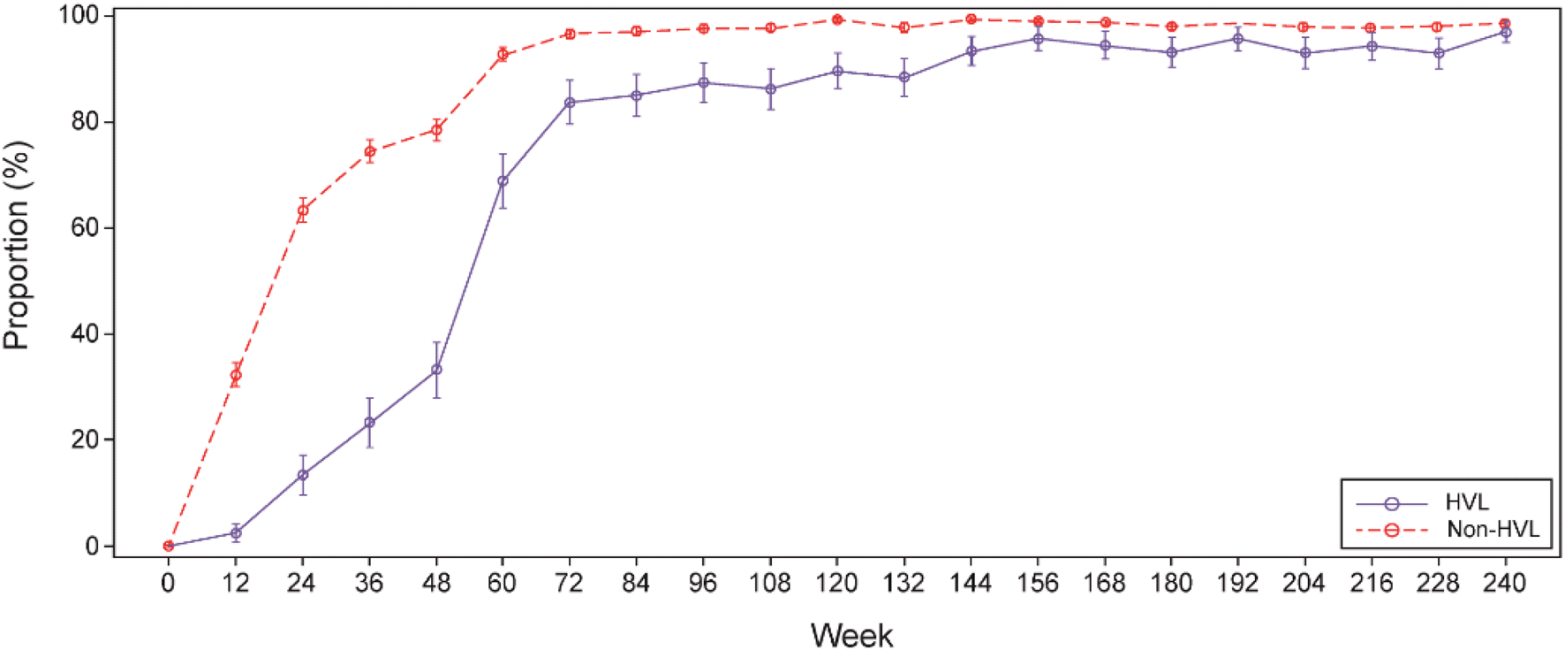
Proportion of HVL and non-HVL patients with HBV DNA < 400 copies/ml over 240 weeks (ITT). HBV, hepatitis B virus; HVL, high virus load; ITT, intent-to-treat. HVL (*n* = 82) and non-HVL (*n* = 427)

Subgroup analyses showed that a smaller proportion of patients in the ITT population achieved HBV DNA < 400 copies/ml at week 48 in the HVL (32.9%) compared with non-HVL (76.8%) group. The difference in proportion of patients with HBV DNA < 400 copies/ml was more pronounced at week 48, but by week 240 the proportion of patients was similar between HVL and non-HVL subgroups (85.4% vs. 88.8%; Fig. 3).

Of 509 patients, 188 (36.9%) had a histology assessment at baseline, of which 182 patients underwent liver biopsy assessment at week 48. Only 27 patients underwent histological assessment at week 240 as majority of the patients elected not to have a repeat liver biopsy assessment performed at this time or at their last study visit. Of these, 19/27 patients showed histological improvement at week 240. Demographic characteristics and baseline disease and on-treatment response characteristics associated with reversal or non-reversal of cirrhosis at year 5 are summarized in Supplementary Table 1. Significant difference was seen in change in platelet from baseline to week 240, Knodell necroinflammatory scores and Knodell fibrosis scores when compared between patients with and without histological improvement. Supplementary Fig. 1 shows Knodell necroinflammatory scores and Knodell fibrosis scores.

### Biochemical and serologic response

At baseline, 98.5% of HBeAg-positive and 87.3% of HBeAg-negative patients had abnormal ALT levels. Similar proportion of patients in the TDF–TDF and ADV–TDF treatment groups had normalized ALT levels at week 240 in HBeAg-positive (80.4% vs. 82.5%) and HBeAg-negative patients (87.5% vs. 84.1%) in the ITT population (Table 2).

In the ITT population, one HBeAg-positive patient had HBsAg loss in the TDF–TDF group without HBsAg seroconversion. None of the HBeAg-negative patients had HBsAg loss or seroconversion at week 240 in the ITT populations. A greater proportion of HBeAg-positive patients in TDF–TDF group compared with ADV–TDF group had HBeAg loss (41.7% vs. 36.4%) and HBeAg seroconversion (32.0% vs. 28.3%) in the ITT population.

The proportions were similar in the PPW population.

The mean reduction in quantitative HBsAg from baseline to week 240 was 0.9 log_10_ IU/mL and 0.7 log_10_ IU/mL in TDF–TDF and ADV–TDF groups, respectively, for HBeAg-positive patients and 0.4 log_10_ IU/mL in both groups for HBeAg-negative patients.

### Resistance surveillance

A summary of mutations during double-blind and open-label phases by treatment in the ITT population is presented in Supplementary Table 2. Sequence changes from baseline in HBV pol/RT during the open-label phase were detected in two patients in TDF–TDF and six patients in ADV–TDF group (Supplementary Table 3). Three patients harbored a reduced mixture of mutations (*n* = 1) or reverted to wild type (*n* = 2). Five patients revealed emerging mutations: rtN/H238Q/K (*n* = 1), rtV224 V/I (*n* = 1), rtG255G/E (*n* = 1), rtM204I + rtL229 V (*n* = 1) and rtM250 M/V + rtL180L/M + rtM204 M/V + rtV173 V/L (*n* = 1). Except for one patient with an emerging mutation of rtG255G/E at week 240, having HBV DNA elevation > 1 log copies/mL from undetectable HBV DNA at week 192, a rapid decline in HBV DNA after detection of emerging mutations was observed in other patients with continued treatment with TDF. No clinical resistance to TDF was observed and no HBV mutations associated with TDF resistance were identified during the study.

### Safety

Overall, the incidence of AEs was similar between the treatment groups (Table 3). The most common AEs were upper respiratory tract infection and increase in ALT levels in TDF–TDF and ADV–TDF groups, respectively. Most of the AEs were mild (43.6% vs. 37.3%) or moderate (9.3% vs. 9.1%) in intensity.

**Table 3:** Summary of adverse events and laboratory abnormalities (safety population)

	TDF–TDF (*N* = 257)	ADV–TDF (*N* = 252)
Any AE	145 (56.4)	130 (51.6)
Drug-related AEs	18 (7.0)	24 (9.5)
Discontinuation due to AE	2 (0.8)	1 (0.4)
Any serious AE	12 (4.7)	20 (7.9)
Deaths	1 (0.4)	0
Frequent AEs (≥ 2% in any group)		
Upper respiratory tract infection	34 (13.2)	25 (9.9)
Increase in alanine aminotransferase	16 (6.2)	8 (3.2)
Hepatic steatosis	10 (3.9)	11 (4.4)
Nasopharyngitis	8 (3.1)	8 (3.2)
Increase in blood CPK	7 (2.7)	8 (3.2)
Renal cyst	6 (2.3)	3 (1.2)
Nephrolithiasis	5 (1.9)	13 (5.2)
Hepatic pain	5 (1.9)	5 (2.0)
Asthenia	4 (1.6)	5 (2.0)
Decrease in blood phosphorus	3 (1.2)	5 (2.0)
Upper abdominal pain	2 (0.8)	6 (2.4)
Any grade 3 or 4 abnormality	56 (21.8)	51 (20.2)
Alanine aminotransferase	19 (7.4)	14 (5.6)
Aspartate aminotransferase	10 (3.9)	6 (2.4)
Prothrombin time	10 (3.9)	17 (6.7)
Creatine kinase	6 (2.3)	4 (1.6)
Neutrophils	6 (2.3)	4 (1.6)
Hemoglobin	4 (1.6)	5 (2.0)
Platelets	4 (1.6)	3 (1.2)
Sodium	2 (0.8)	0
Phosphorus	2 (0.8)	3 (1.2)
Lymphocytes	2 (0.8)	3 (1.2)
Potassium	1 (0.4)	0
Bilirubin	1 (0.4)	1 (0.4)
Glucose	1 (0.4)	2 (0.8)
Amylase	1 (0.4)	2 (0.8)

Data are presented as *n* (%). *ADV* adefovir dipivoxil, *AE* adverse event, *CPK* creatine phosphokinase, *TDF* tenofovir disoproxil fumarate

Serious AEs were reported in 12 (4.7%) patients receiving TDF–TDF and 20 (7.9%) patients receiving ADV–TDF. Two patients (0.8%) developed HCC in the TDF–TDF group, one SAE of synovitis was reported in the patient receiving TDF–TDF and was identified as drug-related study. One death was reported in the TDF–TDF group during the double-blind phase of the study, due to an SAE of malignant glioma and was considered unrelated to study treatment. No deaths were reported during the open-label phase. The overall observed incidence of HCC (0.41% and 1.09%) was lower than rates predicted (1.04% and 2.42%) by the REACH-B model at 3 and 5 years of treatment, respectively. Two AEs of fracture were reported in 1 (0.4%) patient each—ankle fracture in the ADV–TDF group and foot fracture in the TDF–TDF group. The two events were reported as unrelated to study drug treatment.

The frequency of grade 3/4 laboratory abnormalities was similar between TDF–TDF and ADV–TDF groups (Table 3). Grade 3/4 ALT abnormalities were reported in 19 patients in the TDF–TDF group and 14 patients in the ADV–TDF group. No patient had confirmed serum creatinine concentration ≥ 2 mg/dL or CrCl < 50 mL/min. One patient had a confirmed increase of 0.5 mg/dL in serum creatinine from baseline of 0.8 mg/dL to 1.3 mg/dL at week 240 in the TDF–TDF group. The serum creatinine increased during week 96 to week 120 and then decreased to the normal range from week 132 onwards with no change in treatment dose. The patient’s creatinine clearance (CrCl) was below normal range from week 84 up to week 240 and ranged between 64 and 84 mL/min. There were three patients with confirmed grade 3 or 4 phosphorus abnormalities (< 2 mg/dL); all of them were in the TDF–TDF group and had grade 3 phosphorus abnormalities at baseline and reverted to grade 2 or normal range at week 240 with no change in study treatment dose or dose interruption. Among the three patients, one patient was reported to have a medical history of left nephrolithiasis with normal renal function at the study entry. Another patient had normal creatinine clearance of 91 mL/min at baseline, which then slightly declined below the normal range from week 108 up to week 240 and ranged between 72 and 88 mL/min during this period. The third patient did not experience any renal complication and had normal renal function during the entire treatment period.

At baseline, the mean serum creatinine, CrCl and serum phosphorus concentrations among patients with impaired renal function (*n* = 49) were 0.94 mg/dL, 76.9 mL/min and 1.02 mmol/L, respectively, and were stable over 240 weeks (0.98 mg/dL, 71.5 mL/min and 0.99 mmol/L, respectively) (Supplementary Fig. 2). These concentrations were also stable among patients with normal renal function (*n* = 460) (Supplementary Fig. 2).

Among patients with pre-existing HTN (*n* = 9; creatinine 0.87 mg/dL, CrCl 94.6 mL/min and phosphorus 0.93 mmol/L at baseline) or DM (*n* = 5; creatinine 0.80 mg/dL, CrCl 97.8 mL/min and phosphorus 1.01 mmol/L at baseline), the changes in serum creatinine from baseline were small and numerically higher at week 240 in patients with HTN versus DM patients (0.08 mg/dL vs. 0.05 mg/dL). The mean CrCl decreased over time to 82.4 mL/min in patients with HTN or 83.8 mL/min in patients with DM after 240 weeks. The serum phosphorus levels remained stable throughout the treatment period in these patients. No oral phosphate supplement was reported to be administered during TDF treatment in the study.

## Discussion

This 240-week study in patients with chronic HBV infection represents the first publication of long-term experience with TDF in Chinese population. In the present study, viral suppression occurred in majority of both HBeAg-positive and HBeAg-negative patients who received TDF–TDF. The large gap in HBV DNA suppression rates seen at week 48 between the TDF–TDF and ADV–TDF groups was reduced rapidly, when all patients were switched from ADV to receive TDF, a more potent oral antiviral, during the open-label phase and were similar between the treatment groups at the end of 240 weeks. When compared with previous pivotal studies, the overall trend of virological suppression was similar at 48 weeks; however, the suppression was slightly higher in the present study than the suppression observed in previous global phase III pivotal studies [2, 5].

Normalization of ALT was achieved in a similar proportion of patients in the two treatment groups. The proportion of HBeAg-positive patients achieving HBeAg loss or HBeAg seroconversion was consistently higher in the TDF–TDF group versus ADV–TDF group at all major time points through week 240. By week 240, seven patients in the TDF–TDF group and 19 patients in the ADV–TDF group had virological breakthrough. Prompt viral suppression was observed at the follow-up visits in patients with virological breakthrough who had continued treatment with TDF without adding or switching to other antiviral agents. This indicates that continuing TDF treatment with close follow-up is acceptable and feasible even if virological breakthrough has been confirmed. The overall efficacy findings of treatment with TDF in Chinese patients with HBV were similar to those seen in predominantly Caucasian populations in global phase III pivotal studies [2, 5].

Only one HBeAg-positive patient receiving TDF–TDF had HBsAg loss without seroconversion during the 240-week treatment period. This observation is slightly different from the previous studies by Marcellin et al., in which HBsAg loss was observed in 10% of HBeAg-positive patients and 8% of patients had HBsAg seroconversion [6]. This could be explained by the differences in HBV genotypes in both studies. In contrast to the study in Caucasians, who were primarily affected with genotype A and D, the present study had patients with genotype B and C [3, 7].

In a previous global study, long-term suppression of HBV with TDF treatment has resulted in regression of fibrosis and cirrhosis in the majority of patients with chronic HBV [6]. In the present study, among patients who showed histological improvement, the Knodell necroinflammatory and Knodell fibrosis scores were significantly higher compared to patients who did not show improvement. These results are consistent with previous studies, which report that patients with severe necroinflammation and liver fibrosis tend to have greater response [8–10]. It can be explained that sustained viral suppression and inflammation remission are associated with fibrosis regression, and that patients with more severe fibrosis have more prominent beneficial effect of antiviral treatment. However, the sample size with liver biopsy at both baseline and week 240 was limited; we need further study with more data to consolidate this conclusion.

No HBV mutations associated with TDF resistance were identified during both double-blind and open-label phases. These results in Chinese patients are consistent with the TDF profile of a high genetic barrier to the development of resistance, which was observed in a previous global study of TDF in patients with chronic HBV [11].

In subgroups of HVL and non-HVL, the viral suppression at week 48 was considerably lower in HVL patients compared with non-HVL patients. As previously shown, HVL patients took longer time to achieve HBV DNA < 400 copies/ml, the virological response in HVL patients was approaching similar to that of non-HVL patients at week 156 and was maintained until week 240 [12]. Among HVL patients, the virological response in the TDF–TDF group was higher during the first 48 weeks compared with ADV–TDF group; however, viral suppression was achieved in comparable proportion of patients in TDF–TDF and ADV–TDF groups from week 60 up to week 240. Approximately 95% of patients achieved HBV DNA < 400 copies/ml by week 96 in the non-HVL group. This indicated that antiviral efficacy is improved after switching from ADV to TDF; however, a higher level of suppression is achieved earlier when TDF is used as the initial choice for HBV antiviral treatment. These findings are in concordance with the previous studies conducted in predominantly Caucasian and a subgroup of Asian and Pacific Islander patients with HBV [12, 13].

The incidences of AEs were similar in both TDF–TDF and ADV–TDF groups with no new safety concerns in Chinese patients with HBV.

No serious renal AEs occurred in the TDF–TDF group and serum creatinine levels remained stable over 240 weeks of treatment. The CrCl and serum phosphorus levels were also stable in patients with and without renal impairment. The serum creatinine change from baseline for normal renal function was: TDF–TDF: 3.62 ± 6.90 µmol/L; ADV–TDF: 3.35 ± 7.34 µmol/L and impaired renal function: TDF–TDF: 3.91 ± 8.49 µmol/L; ADV–TDF: 3.76 ± 6.56 µmol/L. The CrCl change from baseline for normal renal function was: TDF–TDF: − 9.2 ± 10.75 mL/min; ADV–TDF: − 8.6 ± 13.22 mL/min and impaired renal function: TDF–TDF: − 5.8 ± 8.42 mL/min; ADV–TDF: − 5.6 ± 6.84 mL/min. These findings are consistent with previous controlled studies in which few patients who received TDF demonstrate significant worsening in renal function [14–16], and several real-world cohort studies wherein changes in renal function as determined by eGFR (or CrCl) remained relatively stable over ≥ 3 years of treatment [15–17]. Results from two randomised clinical trials comparing treatment with tenofovir alafenamide (TAF) versus TDF in patients with HBV infection have demonstrated the non-inferiority of TDF over TAF. Although, in comparison with TDF, TAF exhibited lesser toxicity to bone and renal parameters according to the 96-week outcome. However, longer duration of follow-up is required to know about the long-term tendency of renal function change by TDF treatment and interpret the clinical implication of TDF on patients with HBV [18, 19]. In the present study, it was demonstrated that the renal function changed slightly and remained stable in the CHB patients with normal renal function under the continued TDF treatment. The rate of CrCl decreased over time in patients with HTN or DM in both TDF–TDF and ADV–TDF treatment groups. This could have been impacted by aging of patients.

In conclusion, this long-term first-of-its-kind study in Chinese patients with chronic HBV demonstrated that treatment with TDF led to sustained viral suppression without development of resistance for up to 240 weeks in both HBeAg-positive and HBeAg-negative patients. No new safety concerns were found with TDF, making it an effective treatment option in Chinese patients with chronic HBV.

## Electronic supplementary material

Below is the link to the electronic supplementary material.

Supplementary material 1 (DOCX 268 kb)
