# Supplementary material for: Long-term efficacy and safety of tenofovir disoproxil fumarate in Chinese patients with chronic hepatitis B: 5-year results
Source: Hepatol Int. 2019 Apr 11;13(3):260–9. doi: 10.1007/s12072-019-09943-6 (PMC6529393; doi:10.1007/s12072-019-09943-6)
Supplement: Supplementary file 1 — Supplementary material 1 (DOCX 268 kb) [file 12072_2019_9943_MOESM1_ESM.docx]

**Supplementary material:**

**Long-term efficacy and safety of tenofovir disoproxil fumarate in Chinese patients with chronic hepatitis B: 5-year results**

Xieer Liang, Zhiliang Gao, Qing Xie, Jiming Zhang, Jifang Sheng, Jun Cheng, Chengwei Chen, Qing Mao, Wei Zhao, Hong Ren, Deming Tan, Junqi Niu, Shijun Chen, Chen Pan, Hong Tang, Hao Wang, Yimin Mao, Jidong Jia, Qin Ning, Min Xu, Shanming Wu, Jun Li, Xinxin Zhang, Wenyan Zhang, Cui Xiong, Jinlin Hou

**Journal:** Hepatology International

**Corresponding author:**

Jinlin Hou, MD

State Key Laboratory of Organ Failure Research, Guangdong Provincial Key Laboratory of Viral Hepatitis Research, Department of Hepatology Unit and Infectious Diseases, Nanfang Hospital, Southern Medical University, Guangzhou, China;
E-mail: jlhousmu@163.com; Phone: +86-13802727354

**Table of content**

| Table | Table caption | Page number |
| --- | --- | --- |
| Supplementary Table 1 | Comparison between patients with and without histological improvement | 3 |
| Supplementary Table 2 | Summary of mutations (ITT population^†^) | 5 |
| Supplementary Table 3 | Viraemic patients with changes in HBV pol/RT during open-label treatment (ITT Population) | 7 |
| Supplementary Figure 1 | Changes from baseline to Week 240 in the (A) knodell necroinflammatory and (B) fibrosis scores (n=27 with histological assessment) | 8 |
| Supplementary Figure 2 | Mean (A) creatinine, (B) creatinine clearance and (C) phosphorous in patients with impaired and normal renal function (safety population) | 9 |

## Supplementary Table 1. Comparison between patients with and without histological improvement at week 240

| **Characteristics** | **Patients with histological improvement**  **n=19** | **Patients without histological improvement**  **n=8** | **P value** |
| --- | --- | --- | --- |
| Age (years) | 36.2 (9.87) | 38.8 (12.45) | 0.5769 |
| BMI (kg/m^2^) | 23.3 (3.29) | 21.9 (3.58) | 0.3394 |
| Gender, n (%)  Male  Female | 15 (78.95)  4 (21.05) | 8 (100.0)  0 | 0.2855 |
| ALT (U/L) | 158.7 (131.5) | 132.1 (96.39) | 0.6114 |
| Albumin (g/dL) | 45.53 (2.55) | 48.13 (2.80) | 0.0268 |
| Platelet (10^9/L) | 166.1 (44.58) | 175.6 (34.96) | 0.5944 |
| Positive for HBV (months) | 92.19 (67.67) | 183.4 (122.3) | 0.0789 |
| HBV genotype, n (%)  B  BC  C | 6 (31.58)  1 (5.26)  12 (63.16) | 5 (62.50)  0  3 (37.50) | 0.2998 |
| HBV DNA (×${10}^{9}$ copies/mL) | 0.34(0.58) | 1.60 (3.39) | 0.3297 |
| HBsAg value (IU/mL) | 6358.6 (8103.8) | 16293.0 (31327.5) | 0.4042 |
| HBeAg, n (%)  Negative  Positive | 12 (63.16)  7 (36.84) | 5 (62.50)  3 (37.50) | 1.000 |
| Knodell necroinflammatory score | 9.21 (2.32) | 4.75 (2.19) | <0.0001 |
| Knodell fibrosis score | 3.11 (0.94) | 1.13 (0.83) | <0.0001 |
| Previous LAM use, n (%) | 2 (10.53) | 0 | 1.000 |
| HBV DNA <400 copies/mL at Week 240, n (%) | 18 (94.74) | 8 (100.0) | 1.000 |
| ALT normalization at Week 240, n (%) | 16 (94.12)^a^ | 6 (85.71)^b^ | 0.5072 |
| HBeAg loss up to Week 240, n (%) | 3 (42.86)^c^ | 0^d^ | 0.4750 |
| Platelet change from baseline to Week 240, (10^9/L) | 35.39 (18.96)^e^ | 7.13 (22.87) | 0.0030 |
| Albumin change from baseline to Week 240, (g/dL) | 1.42 (3.25) | -0.25 (2.87) | 0.2198 |
| HBeAg change from baseline to Week 240 | -207.4 (374.5) | -448.1 (695.4) | 0.3801 |
| HBsAg loss up to Week 240, n (%) | 0 | 0 | - |
| HBsAg change from baseline to Week 240 (IU/mL) | -3937.2 (8107.8) | -15043.2 (31489.0) | 0.3562 |
| Data presented as mean (SD), unless otherwise specified. ALT, alanine transaminase; BMI, body mass index; HBV, hepatitis B virus; LAM, lamivudine.  ^a^n=17, ^b^n=7, ^c^n=7, ^d^n=3, ^e^n=18.  Reference values of biochemical parameters:  Alanine aminotransferase: 0-48 U/L; Albumin : 32-50 g/L; platelet: 130-400 10^9/L; Creatinine Clearance: males: 85-125 mL/min; females:75-115 mL/min; Phosphate: 0.7-1.45 mmol/L; Serum Creatinine: males: 59.2- 136.1 umol/L; females:42.4-104.3 umol/L | | | |

## Supplementary Table 2. Summary of mutations (ITT population^†^)

|  | **HBV DNA ≥69 IU/mL** | | **Virological breakthrough** | | **Discontinued treatment with viraemia^‡^** | | **Overall** | |
| --- | --- | --- | --- | --- | --- | --- | --- | --- |
|  | **TDF-TDF** | **ADV-TDF** | **TDF-TDF** | **ADV-TDF** | **TDF-TDF** | **ADV-TDF** | **TDF-TDF** | **ADV-TDF** |
| **N** | 257 | 252 | 257 | 252 | 257 | 252 | 257 | 252 |
| **Double-blind phase** | 25 (9.7) | 118 (46.8) | 0 | 6 (2.4) | 0 | 0 | 25 (9.7) | 118 (46.8) |
| Invalid sequence or DNA sequence undetectable | 14 (5.4) | 21 (8.3) | 0 | 0 | 0 | 0 | 14 (5.4) | 21 (8.3) |
| No changes from baseline | 9 (3.5) | 82 (32.5) | 0 | 6 (2.4) | 0 | 0 | 9 (3.5) | 82 (32.5) |
| Wild type^§^ at baseline | 9 (3.5) | 74 (29.4) | 0 | 6 (2.4) | 0 | 0 | 9 (3.5) | 74 (29.4) |
| Documented NA-related^¶^ mutation | 0 | 8 (3.2) | 0 | 0 | 0 | 0 | 0 | 8 (3.2) |
| Sequence changes from baseline | 2 (0.8) | 15 (6.0) | 0 | 0 | 0 | 0 | 2 (0.8) | 15 (6.0) |
| Sequence change at documented NA-related mutation | 2 (0.8) | 6 (2.4) | 0 | 0 | 0 | 0 | 2 (0.8) | 6 (2.4) |
| Sequence change at non-NA-related mutation | 0 | 9 (3.6) | 0 | 0 | 0 | 0 | 0 | 9 (3.6) |
| **Open-label phase** | 16 (6.2) | 17 (6.7) | 3 (1.2) | 6 (2.4) | 1 (0.4) | 2 (0.8) | 17 (6.6) | 21 (8.3) |
| Invalid sequence or DNA sequence undetectable | 5 (1.9) | 7 (2.8) | 0 | 2 (0.8) | 0 | 0 | 5 (1.9) | 9 (3.6) |
| No changes from baseline | 9 (3.5) | 6 (2.4) | 3 (1.2) | 1 (0.4) | 1 (0.4) | 1 (0.4) | 10 (3.9) | 6 (2.4) |
| Wild type^§^ at baseline | 9 (3.5) | 6 (2.4) | 3 (1.2) | 1 (0.4) | 1 (0.4) | 1 (0.4) | 10 (3.9) | 6 (2.4) |
| Documented NA-related^¶^ mutation | 0 | 0 | 0 | 0 | 0 | 0 | 0 | 0 |
| Sequence changes from baseline | 2 (0.8) | 4 (1.6) | 0 | 3 (1.2) | 0 | 1 (0.4) | 2 (0.8) | 6 (2.4) |
| Sequence change at documented NA-related mutation | 1 (0.4) | 2 (0.8) | 0 | 1 (0.4) | 0 | 0 | 1 (0.4) | 2 (0.8) |
| Sequence change at non-NA-related mutation | 1 (0.4) | 2 (0.8) | 0 | 2 (0.8) | 0 | 1 (0.4) | 1 (0.4) | 4 (1.6) |
| Data are presented as n (%). ADV, adefovir dipivoxil; HBV, hepatitis B virus; NA, nucleic acid; TDF, tenofovir disoproxil fumarate.  Of the 25 HBeAg-positive patients qualified for genotypic analysis during the open-label phase, two patients had no resistance surveillance outcome that was recorded as protocol deviation. Of the 19 HBeAg-negative patients qualified for genotypic analysis during the open-label phase, two patients had no resistance surveillance outcome that was recorded as protocol deviation.  ^†^Patients without baseline data for DNA were excluded.  ^‡^Patients who temporarily stopped treatment were excluded.  ^§^Includes patients without sequence changes from consensus and sequence changes from consensus at non-NA-related positions.  ^¶^Includes all widely accepted or frequently reported NA-related mutation:  I. Primary mutations: rtM204I/V/S, rtA181V/T, rtN236T, rtI169T, rtT184S/A/G/I/L, rtS202G/C/I, rtM250V/I/L  II. Secondary compensatory mutations: rtV173L, rtL180M  III. Other reported positions: rtV207I/L, rtS213T, rtV214A/E, rtI233V, rtP237H, rtN238D/S/T | | | | | | | | |

## Supplementary Table 3. Viraemic patients with changes in HBV pol/RT during open-label treatment (ITT Population)

| **S. No** | **Treatment** | **HBeAg status at screening** | **Virological breakthrough (Yes/No)** | **Visit** | **HBV DNA (log_10_ copies/mL)** | **Confirmed mutation** | **Non-confirmed mutation** |
| --- | --- | --- | --- | --- | --- | --- | --- |
| 1. | TDF-TDF | Negative |  | Baseline | 5.31 | S213T, V173L/V, M204V/M, L180M/L | - |
|  |  |  | No | Week 96 | 2.62 | V173L, M204V, S213T | - |
| 2. | TDF-TDF | Positive |  | Baseline | 9.2 | Wild type | - |
|  |  |  | No | Week 144 | 2.83 | Wild type | N/H238Q/K |
| 3. | ADV-TDF | Negative |  | Baseline | 7.21 | Wild type | - |
|  |  |  | No | Week 96 | 3.2 | M204I | L229V |
| 4. | ADV-TDF | Negative |  | Baseline | 9.31 | Wild type | - |
|  |  |  | Yes | Week 144 | 2.95 | M250M/V, L180L/M, M204M/V, V173V/L | - |
| 5. | ADV-TDF | Negative |  | Baseline | 7.09 | Wild type | A223A/S, V224V/I |
|  |  |  | Yes | Week 204 | 3.54 | Wild type | - |
| 6. | ADV-TDF | Negative |  | Baseline | 7.3 | Wild type | A223S/A |
|  |  |  | Yes | Week 216 | 4.01 | Wild type | A223S/A, V224V/I |
| 7. | ADV-TDF | Positive |  | Baseline | 8.01 | Wild type | A222T |
|  |  |  | Yes | Week 240 | 3.22 | Wild type | A222A/T |
| 8. | ADV-TDF | Negative |  | Baseline | 7.98 | Wild type | - |
|  |  |  | Yes | Week 240 | 3.48 | Wild type | G255G/E |
| ADV, adefovir dipivoxil; HBV, hepatitis B virus; pol/RT, polymerase-reverse transcriptase; TDF, tenofovir disoproxil fumarate. | | | | | | | |

**Supplementary Figure 1.** **Changes from baseline to Week 240 in the (A) knodell necroinflammatory and (B) fibrosis scores (n=27 with histological assessment)**

1. B)


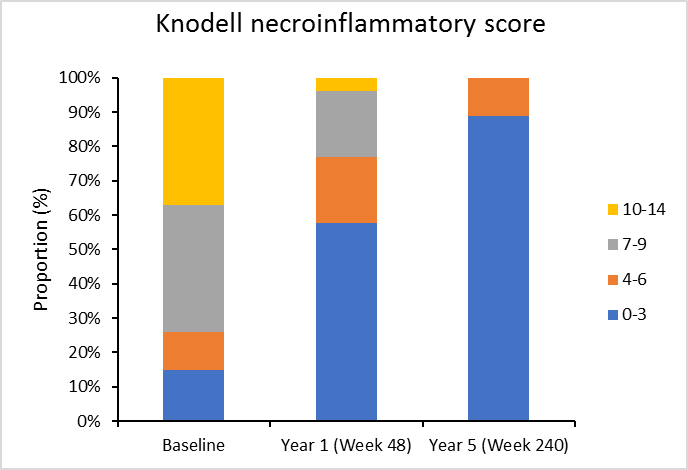

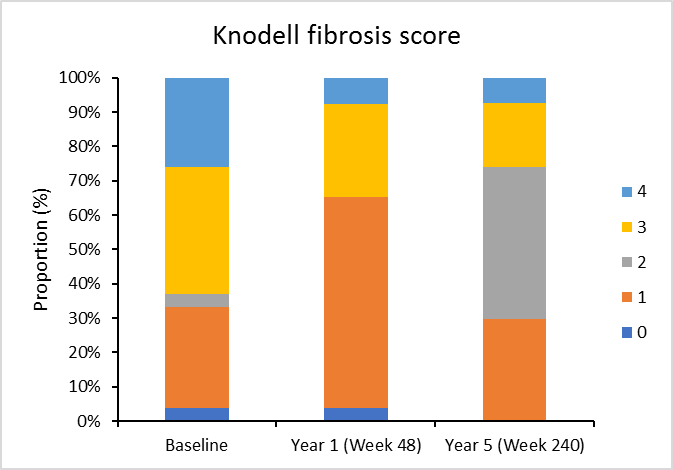


**Supplementary Figure 2. Mean (A) creatinine, (B) creatinine clearance and (C) phosphorous in patients with impaired and normal renal function (safety population)**


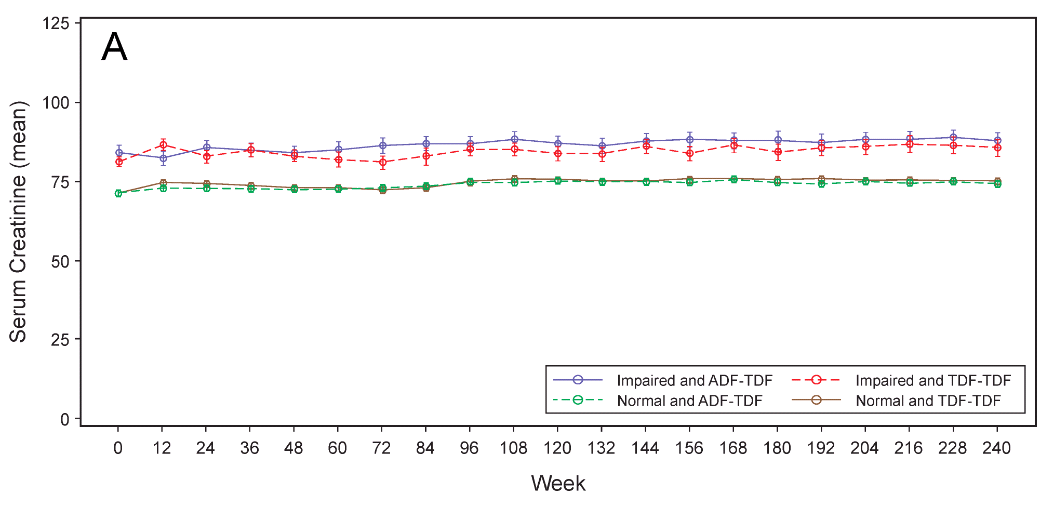


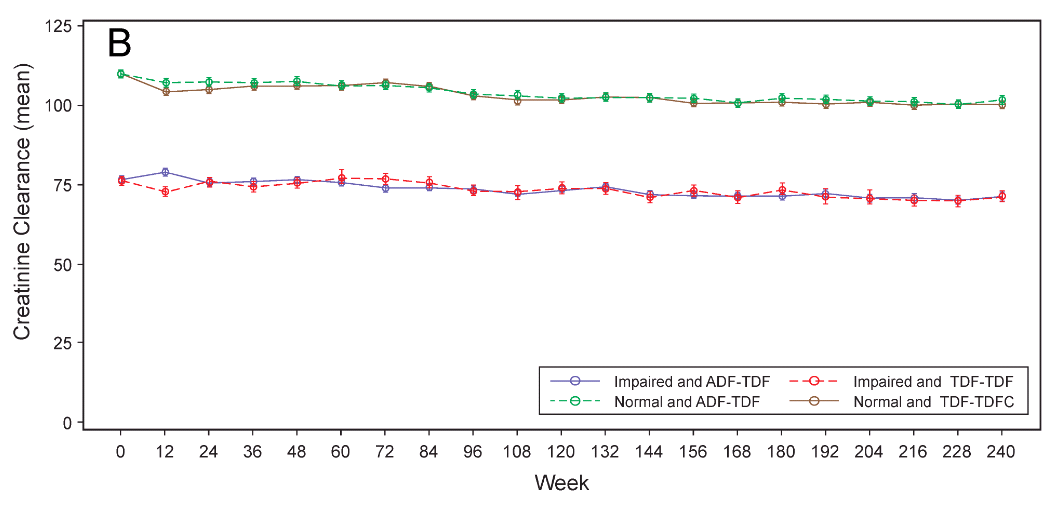


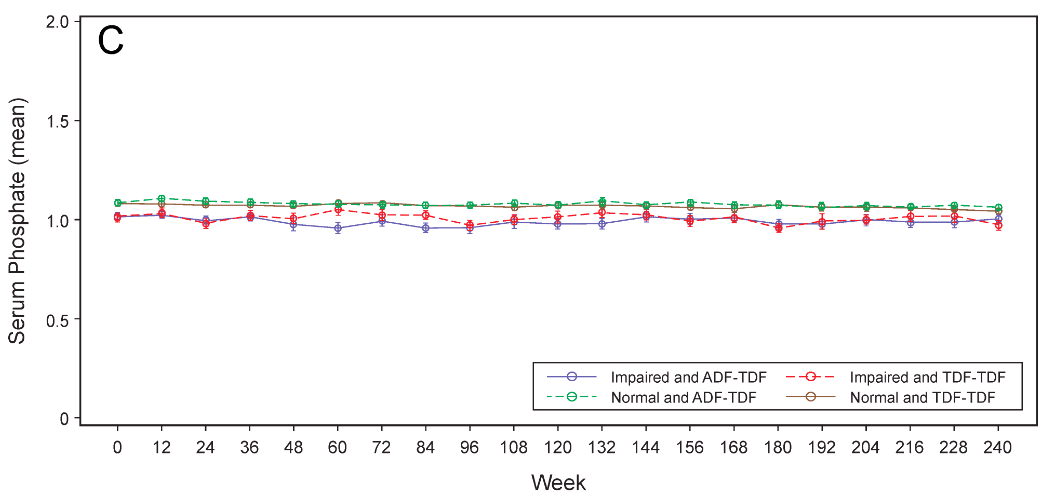


ADV, adefovir dipivoxil; TDF, tenofovir disoproxil fumarate.

|  | **TDF-TDF** | **ADV-TDF** |
| --- | --- | --- |
| **serum creatinine change from baseline:** |  |  |
| Normal renal function: | 3.62 ± 6.90 | 3.35 ± 7.34 |
| impaired renal function: | 3.91 ± 8.49 | 3.76 ± 6.56 |
| **creatinine clearance change from baseline:** |  |  |
| Normal renal function | -9.20 ± 10.75 | -8.60 ± 13.22 |
| impaired renal function | -5.80 ± 8.42 | -5.60 ± 6.84 |
